# Interpretable machine learning enables early identification of financial risk and resource optimization in colorectal cancer surgery

**DOI:** 10.3389/fpubh.2026.1918534

**Published:** 2026-08-14

**Authors:** Biao Fan, Zihao Bian, Jing Wang, Zhiyi Luo, Weiqing Xiong, Ning Zhao, Zeyu Zhang, Lingqian Wang, Shuyuan Cheng, Zongjiu Zhang

**Affiliations:** 1 Institute for Hospital Management, Tsinghua University, Beijing, China; 2 Key Laboratory of Carcinogenesis and Translational Research (Ministry of Education/Beijing), Gastrointestinal Cancer Center, Peking University Cancer Hospital & Institute, Beijing, China; 3 Key Laboratory of Carcinogenesis and Translational Research (Ministry of Education/Beijing), Endoscopy Center, Peking University Cancer Hospital & Institute, Beijing, China; 4 School of Biomedical Engineering, Tsinghua University, Beijing, China; 5 The First Affiliated Hospital of Sun Yat-sen University, Guangzhou, Guangdong, China

**Keywords:** clinical decision support, colorectal cancer, diagnosis-related groups, explainable machine learning, financial risk prediction, resource allocation, value-based cancer care

## Abstract

**Background:**

Colorectal cancer (CRC) imposes major clinical and economic pressure on health systems. Under diagnosis-related group (DRG) payment, fixed reimbursement benchmarks may not capture the heterogeneity of surgical oncology or the resources required by patients with complex disease, exposing hospitals to financial losses and potentially affecting equitable access to high-quality cancer care. We developed an interpretable machine learning framework to estimate DRG-related financial risk in CRC surgery and support perioperative clinical review and local resource planning.

**Methods:**

We retrospectively reviewed 2,081 patients who underwent CRC surgery between 2014 and 2024. Candidate features were screened for multicollinearity, assessed using Boruta, and refined through clinical review. Synthetic minority oversampling was then applied to reduce class imbalance. Six machine learning algorithms were trained with cross-validation and hyperparameter tuning. The best-performing model was interpreted using SHapley Additive exPlanations (SHAP), and Sequential Forward Selection was used to reduce inputs to a clinically feasible subset. These variables were incorporated into an interactive web-based tool for individualized risk assessment. Calibration plots, Brier score, and decision curve analysis were used to evaluate reliability and clinical net benefit. A prospective cohort of 12 patients tested workflow feasibility during routine perioperative assessment.

**Results:**

DRG-related financial risk, defined as expenditure above the DRG benchmark, was present in 38.4% of patients in the retrospective cohort. The random forest model achieved the best discrimination, with an area under the receiver operating characteristic curve of 0.852. SHAP analysis identified surgical approach, postoperative complications, length of hospital stay, and comorbidity burden as major contributors to predicted financial risk. The simplified model remained well calibrated and showed meaningful net benefit on decision curve analysis. In the prospective phase, clinicians used the web-based tool to stratify individual risk and simulate modifiable perioperative strategies for pathway optimization in real clinical workflow.

**Conclusion:**

An interpretable machine-learning framework can provide individualized estimation of DRG-related financial risk in CRC surgery. Its integration into a point-of-care decision-support tool may support perioperative risk review, resource planning, and complex-case management under DRG-based reimbursement.

## 1 Introduction

Colorectal cancer (CRC) remains a major contributor to the global cancer burden. In 2022, the International Agency for Research on Cancer reported approximately 1.92 million new CRC cases, equivalent to 10.2% of all malignant tumor diagnoses (1). CRC was therefore ranked third in incidence and second in cancer-related mortality worldwide. A similar pattern has been reported in China, where the latest National Cancer Center of China report placed CRC second for incidence and fourth for mortality (2). These data indicate that CRC remains among the five leading malignancies at both regional and global levels (3). Because management commonly includes diagnostic evaluation, surgery, postoperative recovery, and supportive care, the disease also generates substantial demand for hospital resources.

The financial implications of CRC parallel its clinical burden (4). Direct treatment costs are substantial for hospitals, and patients and families may incur additional non-medical expenses in both developed and developing settings (5–7). Diagnosis-Related Group (DRG) payment was introduced to improve cost control and reimbursement standardization. However, standardized payment does not fully account for the heterogeneity of surgical oncology. Patients with advanced disease, severe comorbidity, or technically difficult operations may require care that exceeds the fixed benchmark. When such resource use is clinically necessary, hospitals remain exposed to DRG-related financial risk (8).

The gap between necessary care and fixed reimbursement may affect both institutional sustainability and patient access. Repeated financial losses can weaken hospital capacity, whereas strong cost pressure may reduce willingness to accept complex cases. This risk is particularly relevant for vulnerable patients when hospitals anticipate reimbursement shortfalls (9). Early perioperative estimation of financial risk can therefore support earlier resource planning and provide quantitative evidence for exceptional reimbursement, including special case negotiations. In this setting, prediction is intended to protect clinically necessary care by making financial exposure visible before treatment decisions and administrative review occur.

Earlier cost studies have generally relied on conventional statistical methods to identify clinical determinants (10, 11). Those approaches remain informative, but they are less suited to high-dimensional predictors and non-linear interactions among clinical risks. Machine learning (ML) has been used to predict economic outcomes in other diseases (12–14), whereas CRC resource-management applications remain relatively sparse. A further difficulty is implementation: many models are hard for clinicians to interpret and are not translated into point-of-care decision support. As a result, their value for routine public health administration and proactive hospital management is limited. For practical use, a model must show why risk changes and present that information in a form that can guide action.

This study was designed to bridge computational modeling and public health practice by developing an interpretable ML framework for DRG-related financial risk in CRC surgery. To the best of our knowledge, previous CRC financial-risk studies have not combined an interactive point-of-care web tool with prospective feasibility assessment in a real-world clinical workflow. Using retrospective data from 2,081 patients, we trained the predictive model and used SHapley Additive exPlanations (SHAP) with Sequential Forward Selection (SFS) to retain clinically relevant factors while reducing data-entry burden. The tool was subsequently applied in 12 real-world cases to explore whether risk estimates could support perioperative pathway review rather than retrospective cost accounting alone.

## 2 Methods

### 2.1 Study participants

**Figure 1:**
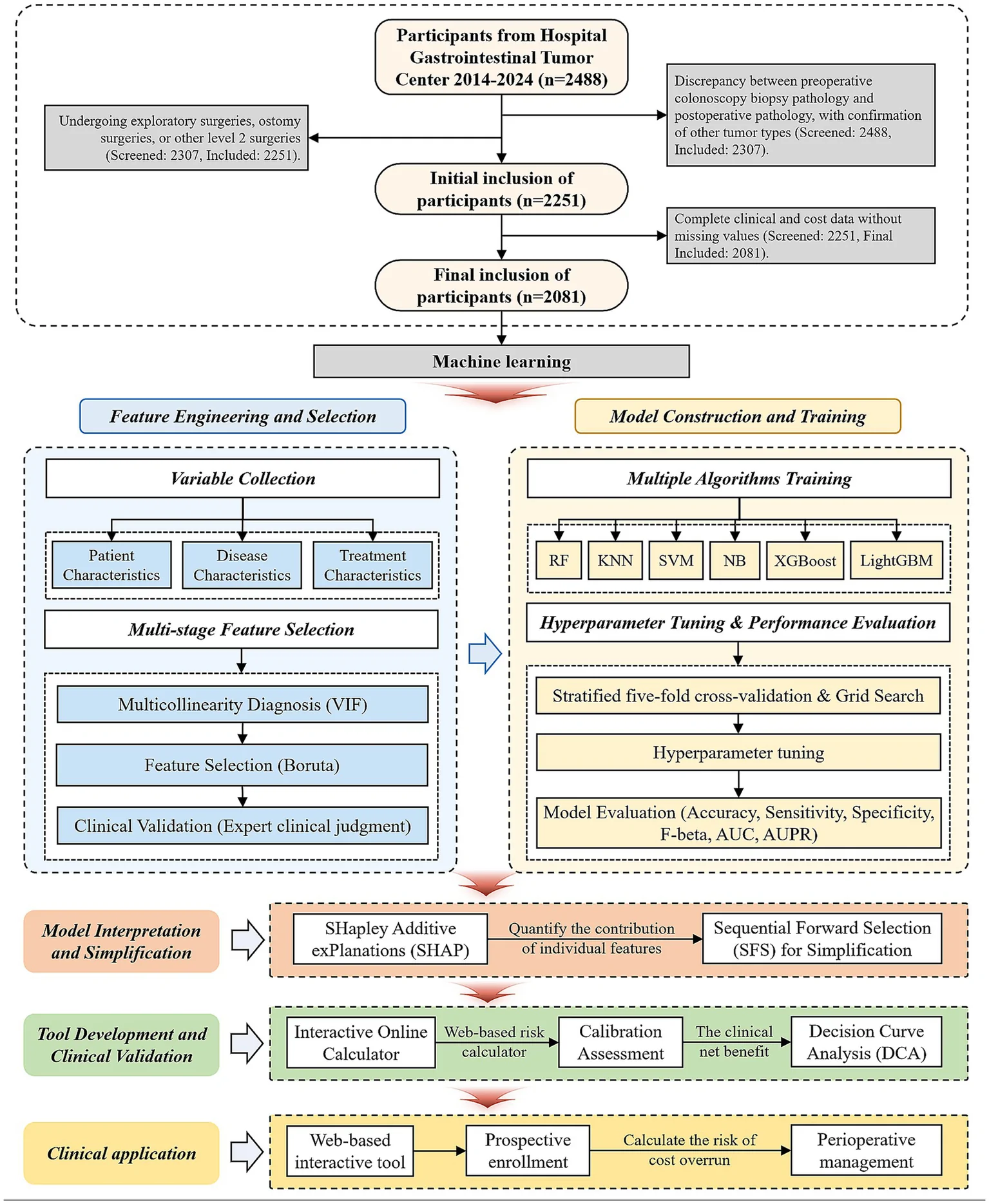
Inclusion of study participants and main analytical approach.

Patients were identified from the Gastrointestinal Oncology Center of Peking University Cancer Hospital, covering admissions for colorectal cancer surgery from October 2014 to October 2024. Inclusion required perioperative pathological confirmation of colorectal adenocarcinoma or signet ring cell carcinoma, a level 3 or 4 operation under DRG 2.0 GB39, and complete clinical and financial records. Cases were excluded when postoperative pathology indicated a different tumor type despite the preoperative colonoscopy biopsy, including neuroendocrine tumor and lymphoma. Exploratory procedures, ostomy procedures, and other level 2 operations were also excluded because their treatment pathways and resource-use patterns differed from the target cohort. After screening, 2,081 inpatients were included. Demographic, disease, treatment, and economic variables were collected using consistent definitions across the study period. The cohort satisfied the Events Per Variable (EPV) requirement for the planned multivariate analysis (15), and the selection process is summarized in Figure 1.

### 2.2 Covariate collection

Candidate predictors were organized according to their position in the care pathway. Patient-level information included gender, age, health insurance modality, comorbidity type and number (categorized as 0, 1, or ≥2), surgical history, smoking, alcohol consumption, drug allergy history, and perioperative hemoglobin, albumin, and albumin-to-globulin ratio. Disease-level information included tumor location, pathological type, differentiation grade, maximum tumor diameter, multifocality, and TNM stage. Treatment-level information included neoadjuvant chemotherapy, number of hospitalizations (categorized as 1 or ≥2), length of hospital stay (categorized as ≤10 or >10 days), surgical approach, radicality of resection, combined organ resection, and postoperative complications. Organizing the variables in these three groups allowed baseline vulnerability, tumor complexity, and treatment-related resource use to be interpreted separately. Full definitions and category specifications are reported in Supplementary Table S1.

### 2.3 Calculation of medical resource consumption

DRG-related financial risk was the primary endpoint, defined as adjusted total hospitalization expenditure exceeding the fixed DRG benchmark. Clinical interpretation of benchmark exceedance considered case complexity and the documented resource requirements of care. Total hospitalization expenditure included direct costs for diagnostic services, consumables, pharmaceuticals, surgery, and nursing care; the detailed components are provided in Supplementary Table S2. Because the cohort spanned 2014–2024, historical expenditures were adjusted to 2024 Beijing medical-service price levels using annual CPI ratios for Medical Services in Beijing (Supplementary Table S3). Each annual CPI was expressed with the preceding year set to 100. Expenditure recorded in year y was converted using the cumulative product of annual CPI ratios from year y + 1 through 2024, while expenditure recorded in 2024 was unchanged. The 2024 Beijing DRG 2.0 GB39 benchmark was then used as a common policy reference to define the endpoint consistently across the retrospective cohort. This approach aligned model development with the current reimbursement framework and allowed cases from different calendar years to be compared under the same benchmark. Adjusted expenditure above CNY 99,900 was coded as 1 (above-benchmark group), whereas expenditure at or below the benchmark was coded as 0 (within-benchmark group).

## 3 Statistical analysis

### 3.1 Baseline statistical comparison

We summarized the study population using frequencies and percentages according to the categories shown in Table 1. The grouping thresholds were predefined for descriptive analysis based on clinically interpretable cutoffs and institutional practice. Group comparisons were performed using the Chi-square test or Fisher’s exact test, as appropriate. A two-sided *p*-value below 0.05 was considered statistically significant. Baseline analyses were performed in R software (version 4.4).

**Table 1:** Cross-tabulation analysis of DRG-related financial risk based on the DRG 2.0 benchmark and patient characteristics.

Variable	Category	Within DRG Benchmark (*n* = 1,282), *n* (%)	Above DRG Benchmark (*n* = 799), *n* (%)	*P*-value
Baseline characteristics				
Sex	Male	774 (60.6)	517 (64.7)	0.048
	Female	508 (39.6)	282 (35.3)	
Age (years)	<55	381 (29.7)	192 (24.0)	<0.001
	55–64	478 (37.3)	258 (32.3)	
	65–74	331 (25.8)	276 (34.5)	
	≥75	92 (7.2)	73 (9.1)	
Insurance type	Beijing urban	338 (26.4)	200 (25.0)	0.065
	Beijing rural	2 (0.2)	3 (0.4)	
	Non-Beijing urban	823 (64.2)	547 (68.5)	
	Non-Beijing rural	109 (8.5)	44 (5.5)	
	Self-pay	10 (0.8)	5 (0.6)	
Number of comorbidities	0	677 (52.8)	333 (41.7)	<0.001
	1	389 (30.3)	280 (35.0)	
	≥2	216 (16.8)	186 (23.3)	
History of surgery	Yes	666 (52.0)	401 (50.2)	0.434
	No	616 (48.0)	398 (49.8)	
Smoking history	Yes	507 (39.5)	370 (46.3)	0.002
	No	775 (60.5)	429 (53.7)	
Alcohol consumption	Yes	342 (26.7)	227 (28.4)	0.388
	No	940 (73.3)	572 (71.6)	
Drug allergy history	Yes	120 (9.4)	62 (7.8)	0.209
	No	1,162 (90.6)	737 (92.2)	
Hemoglobin (g/L)	<110	243 (19.0)	152 (19.0)	0.982
	≥110	1,037 (81.0)	647 (81.0)	
Albumin (g/L)	<35	133 (10.4)	95 (11.9)	0.234
	35–45	790 (61.8)	506 (63.3)	
	≥45	356 (27.8)	198 (24.8)	
A/G ratio	<1.0	6 (0.5)	11 (1.4)	0.031
	1.0–2.5	1,245 (97.3)	777 (97.2)	
	≥2.5	29 (2.3)	11 (1.4)	
Disease characteristics				
Tumor location	Right colon	305 (23.8)	140 (17.5)	<0.001
	Left colon	429 (33.5)	239 (29.9)	
	Rectum	546 (42.6)	415 (51.9)	
	Multiple sites	2 (0.2)	5 (0.6)	
Pathological type	Adenocarcinoma	1,166 (91.0)	728 (91.1)	0.559
	Mucinous adenocarcinoma	83 (6.5)	55 (6.9)	
	Signet-ring cell carcinoma	24 (1.9)	14 (1.8)	
	Others	9 (0.7)	2 (0.3)	
Differentiation	Well	40 (3.1)	24 (3.0)	0.460
	Moderate	1,036 (80.8)	647 (81.0)	
	Poor	187 (14.6)	121 (15.1)	
	Undifferentiated	7 (0.5)	5 (0.6)	
	Unknown	12 (0.9)	2 (0.3)	
Tumor size (cm)	≤3	537 (41.9)	332 (41.6)	0.813
	3–5	472 (36.8)	288 (36.0)	
	>5	272 (21.2)	179 (22.4)	
Multiple tumors	Yes	13 (1.0)	16 (2.0)	0.061
	No	1,269 (99.0)	783 (98.0)	
T stage	T0/Tis	71 (5.5)	35 (4.4)	0.372
	T1	73 (5.7)	49 (6.1)	
	T2	165 (12.9)	101 (12.6)	
	T3	801 (62.5)	485 (60.7)	
	T4	172 (13.4)	129 (16.1)	
N stage	N0	701 (54.7)	395 (49.4)	0.112
	N1	361 (28.2)	242 (30.3)	
	N2	219 (17.1)	161 (20.2)	
	N3	1 (0.1)	1 (0.1)	
M stage	M0	1,172 (91.4)	672 (84.1)	<0.001
	M1	110 (8.6)	127 (15.9)	
Treatment characteristics				
Preoperative antitumor therapy	Yes	288 (22.5)	236 (29.5)	<0.001
	No	994 (77.5)	563 (70.5)	
Number of hospitalizations	1	1,003 (78.2)	559 (70.0)	<0.001
	≥2	279 (21.8)	240 (30.0)	
Length of stay (days)	≤10	695 (54.2)	291 (36.4)	<0.001
	>10	587 (45.8)	508 (63.6)	
Postoperative complications	Yes	49 (3.8)	184 (23.0)	<0.001
	No	1,233 (96.2)	615 (77.0)	
Radicality of surgery	Curative resection	1,158 (90.3)	694 (86.9)	0.014
	Palliative resection	124 (9.7)	105 (13.1)	
Surgical approach	Open	331 (25.8)	103 (12.9)	<0.001
	Laparoscopy-assisted	442 (34.5)	511 (64.0)	
	Laparoscopic	509 (39.7)	185 (23.2)	
Combined organ resection	Yes	2 (0.2)	6 (0.8)	0.033
	No	1,280 (99.8)	793 (99.2)	

### 3.2 Feature selection in machine learning analysis

Feature selection was handled as a combined statistical and clinical process after the cohort had been divided into a development dataset and an independent testing set. Within the development data, we began by calculating the Variance Inflation Factor (VIF), excluding variables only if the VIF exceeded 3. Next, the Boruta algorithm, run within a Random Forest framework, repeatedly assessed feature importance and marked variables with a confirmed association with the outcome (16, 17). The statistical shortlist was then reviewed by clinical experts for medical plausibility. To reduce the imbalance between outcome classes, SMOTE was applied only within the development data. Continuous variables were standardized with a Standard Scaler, with scaling parameters fitted in the development data and then applied to the independent testing set. This sequence removed redundancy before feature ranking and kept algorithmic selection anchored to clinical reasoning. Standardization was performed after the feature set had been defined so that continuous inputs entered the classifiers on comparable scales.

### 3.3 Model construction and performance evaluation

Six algorithms were compared for binary classification of DRG-related financial risk in CRC: Random Forest (RF), K-Nearest Neighbors (KNN), Light Gradient Boosting Machine (LightGBM), Support Vector Machine (SVM), Naive Bayes (NB), and Extreme Gradient Boosting (XGBoost). Model inputs consisted of 26 variables, including patient characteristics (*n* = 11), disease characteristics (*n* = 8), and treatment characteristics (*n* = 7). We randomly assigned 80% of the cohort to model development and reserved the remaining 20% as an independent testing set. Hyperparameters were tuned only within the development data by Grid Search with stratified five-fold cross-validation; the testing set was kept separate throughout. Performance was described with Accuracy, Sensitivity, Specificity, F-beta score, Area Under the Receiver Operating Characteristic Curve (AUC), and Area Under the Precision-Recall Curve (AUPR) (18). Because class imbalance can make any single metric misleading, the best overall model was advanced to the interpretation and deployment stages. To examine model robustness to the inclusion of proximal resource-use indicators, we performed a sensitivity analysis excluding postoperative complications and length of hospital stay; detailed results are provided in Supplementary Table S5.

### 3.4 Model interpretability and web-tool deployment

The final model was interpreted with SHAP. At the cohort level, SHAP values indicated which variables contributed most to prediction; at the patient level, they showed whether each feature increased or decreased the estimated DRG-related financial risk (19). This ordering was then used as the starting sequence for Sequential Forward Selection (SFS). Variables were added step by step until extra inputs no longer improved performance meaningfully, producing a smaller feature set with optimal predictive accuracy (20). SHAP-guided ordering and SFS were performed within the development data before the final reduced model was evaluated on the independent testing set. Those selected variables were incorporated into a web-based calculator that could return a patient-specific risk estimate during clinical use. Agreement between predicted and observed probabilities was evaluated with a calibration curve and Brier score. Decision Curve Analysis (DCA) compared net benefit across threshold probabilities with treat-all and treat-none strategies (21). The same workflow therefore linked interpretation, simplification, and clinical evaluation.

### 3.5 Prospective evaluation in real-world scenarios

We next examined whether the deployed web tool could be used in routine perioperative workflow. An independent prospective cohort of 12 inpatients admitted for CRC surgery was assessed with the model during perioperative assessment. When the tool identified high DRG-related financial risk, clinicians used the interactive interface to adjust modifiable clinical variables and observe the simulated change in risk. The clinical team then considered individualized perioperative interventions on the basis of these simulations. Postoperative expenditure and clinical outcomes were collected prospectively. This feasibility-oriented design allowed us to examine integration of the tool into perioperative workflow and to document how risk estimates were reviewed during clinical management.

## 4 Results

### 4.1 Characteristics of the study population

After eligibility screening, 2,081 patients were retained for the final analysis. DRG-related financial risk, defined as adjusted hospitalization expenditure above the DRG benchmark, was present in 38.4% of the cohort (799/2,081). Table 1 shows that the above-benchmark and within-benchmark groups differed across several clinical dimensions. For patient characteristics, the above-benchmark group was older (*p* < 0.001), had a greater comorbidity burden (*p* < 0.001), and more often had a smoking history (*p* = 0.002). Disease characteristics also differed: rectal tumors (*p* < 0.001) and distant metastasis (M1 stage, *p* < 0.001) were more common in the above-benchmark group. The largest gaps appeared in treatment-related factors. Preoperative antitumor therapy (*p* < 0.001) and laparoscopy-assisted surgery (*p* < 0.001) were more frequent in the above-benchmark group. Resource-use measures followed the same pattern, including length of stay beyond 10 days (63.6% vs. 36.4%, *p* < 0.001) and multiple hospitalizations (*p* < 0.001). Postoperative complications were recorded in 23.0% of the above-benchmark group and 3.8% of the within-benchmark group (*p* < 0.001). Supplementary Table S4 provides the detailed correlations between patient features and resource consumption variables, indicating where excess use was concentrated.

### 4.2 Feature selection for machine learning

**Figure 2:**
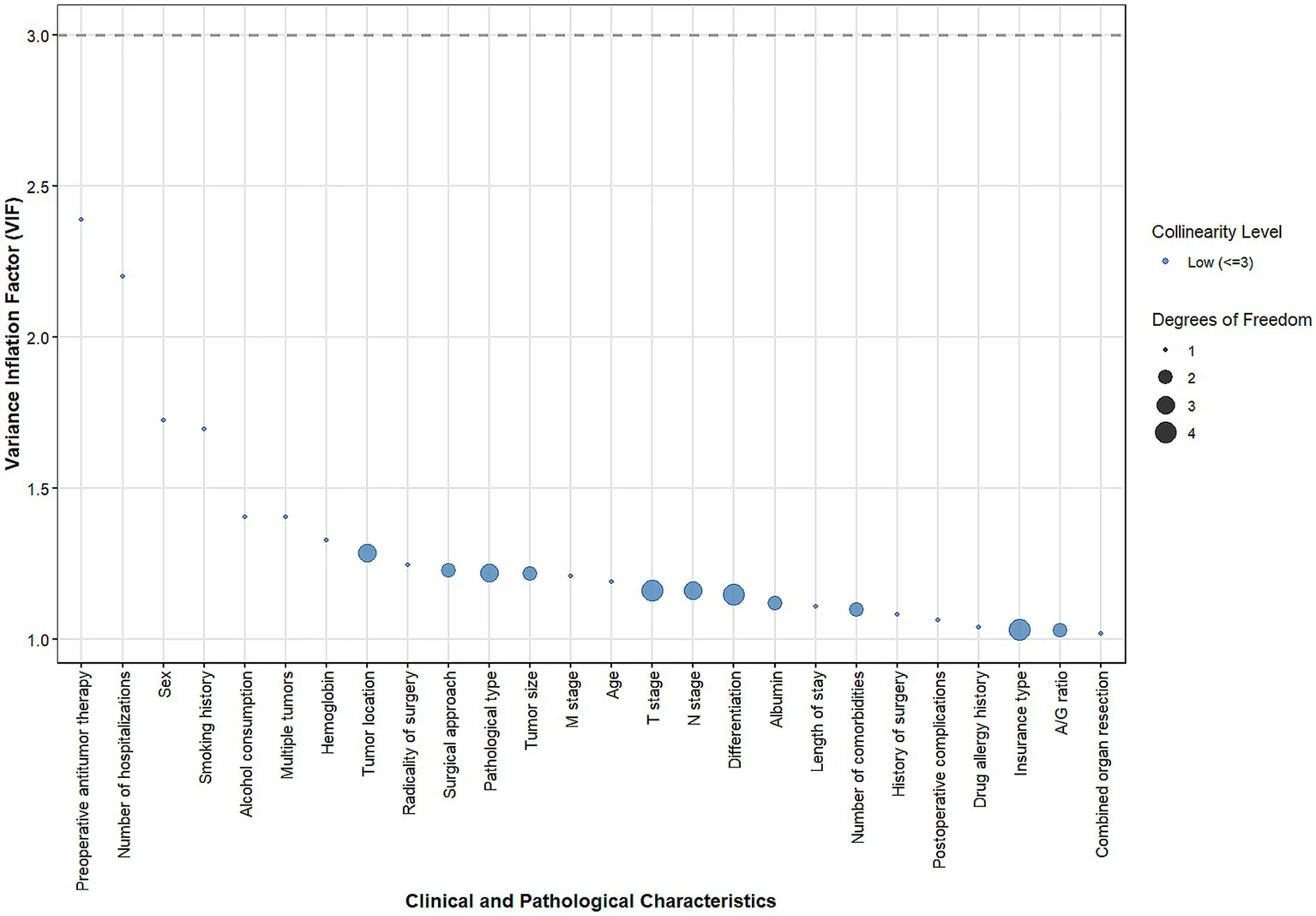
Results of variance inflation factor (VIF) analysis for multicollinearity diagnostics.

**Figure 3:**
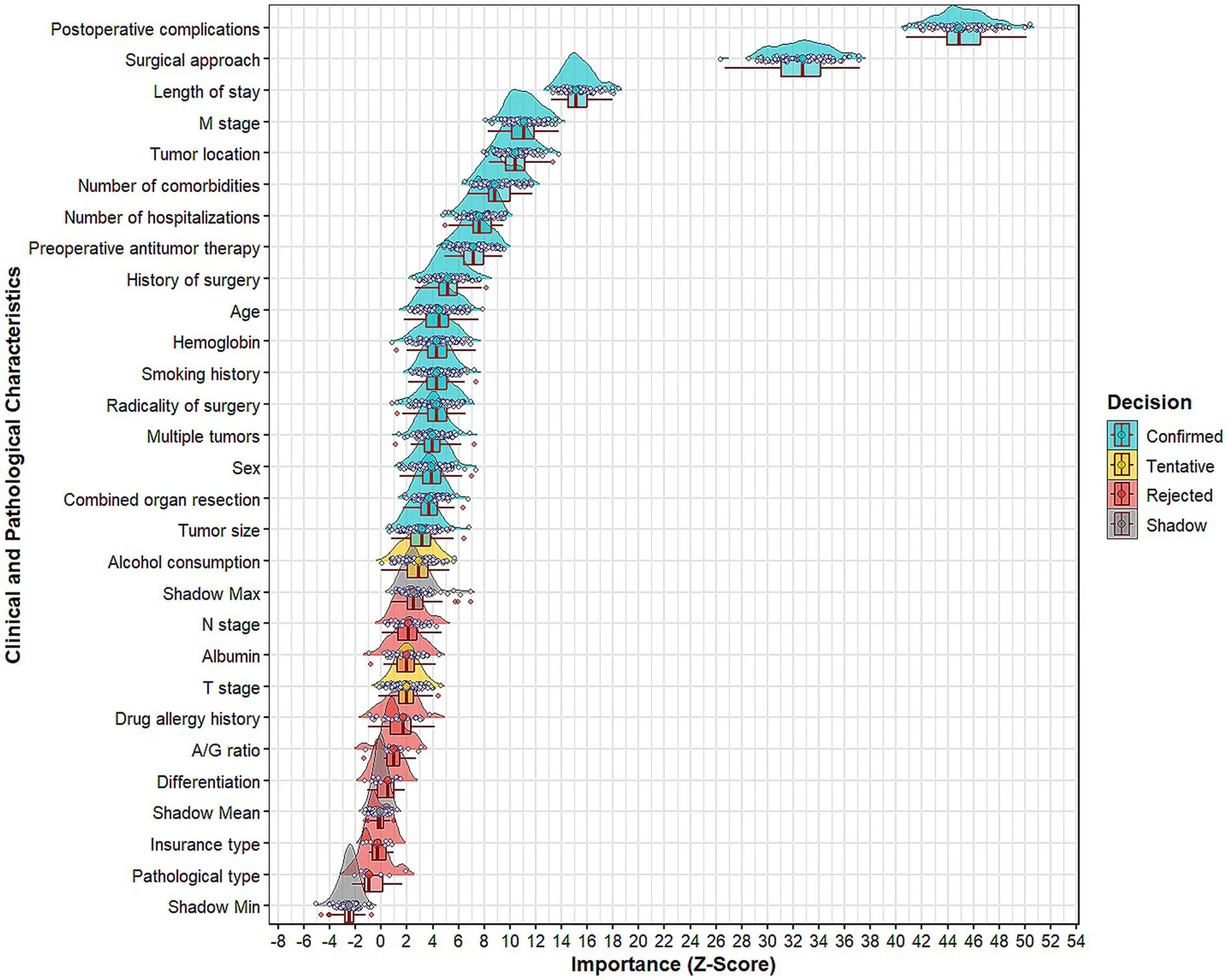
Results of Boruta feature selection.

Feature selection was performed in three stages. First, multicollinearity was assessed across the 26 candidate variables; all VIF values were below 3 (Figure 2), and no variable was excluded on this basis. Second, Boruta was applied to the remaining variables and identified 19 predictors as “confirmed” for DRG-related financial risk (Figure 3). Third, the algorithmic shortlist was evaluated by clinicians for consistency with patient status, tumor biology, and the surgical pathway. The final model inputs therefore reflected both data-driven screening and clinical judgment. Their coverage of demographic, pathological, and operative information also helped link subsequent predictions to recognizable clinical features.

### 4.3 Performance evaluation of machine learning models

**Figure 4:**
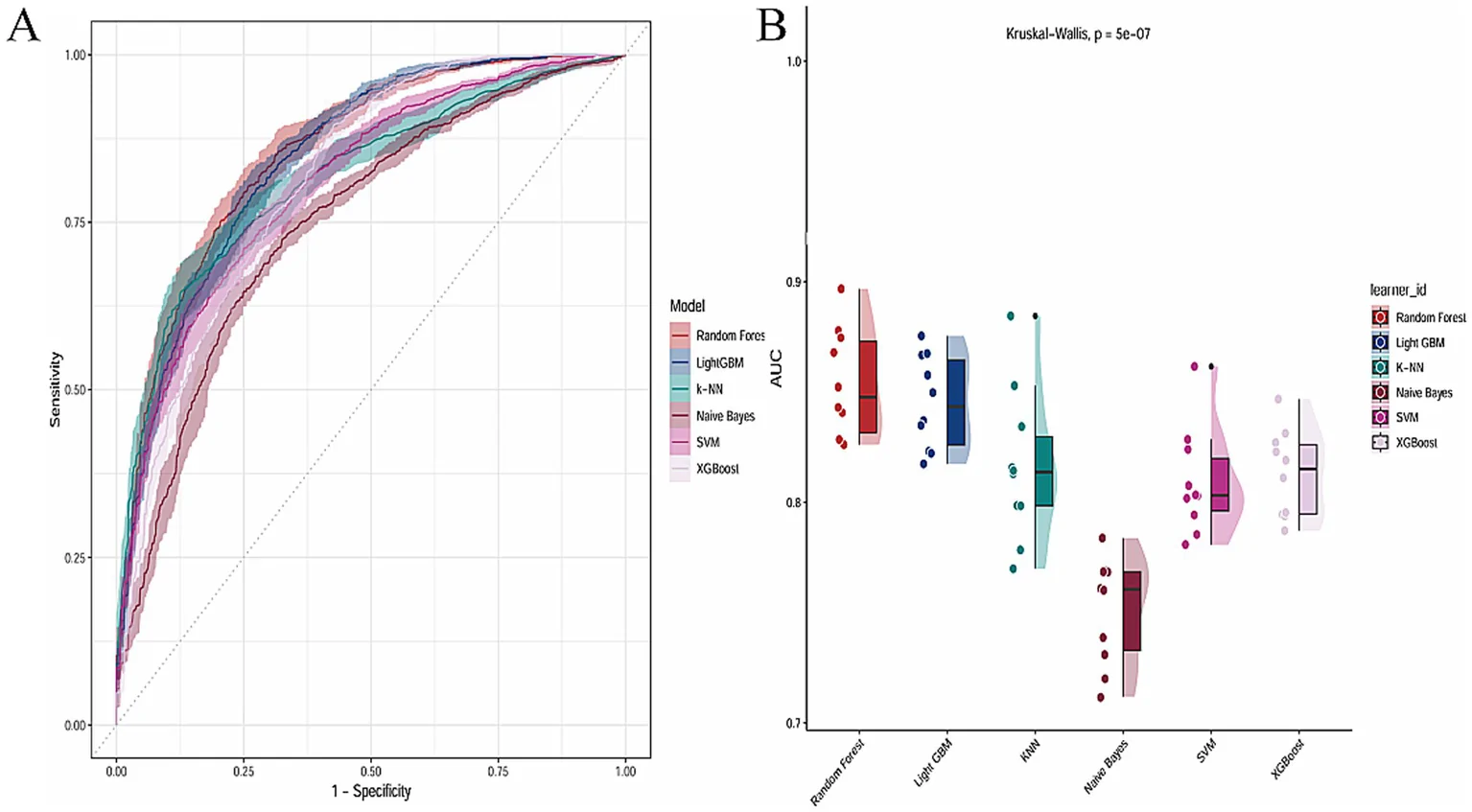
ROC analysis of six machine learning models for predicting DRG-related financial risk. **(A)** Receiver operating characteristic (ROC) curves of the six models. **(B)** Area under the ROC curve (AUC) values for each model.

Testing-set performance for the six machine learning algorithms is shown in Table 2 and Figure 4 (RF, KNN, LightGBM, SVM, NB, and XGBoost). The Random Forest (RF) model ranked highest overall, achieving the largest Area Under the Curve (AUC = 0.852), an Accuracy of 0.768, and an F-beta score of 0.772. We considered both discrimination and the balance between sensitivity and specificity across the main outcome classes. The gradient-boosting ensembles also performed well, with LightGBM (AUC = 0.841) and XGBoost (AUC = 0.835) showing robust predictive capacity, but RF provided the most favorable sensitivity-specificity trade-off. For that reason, Random Forest was selected for the subsequent interpretable analysis and for implementation in the clinical decision tool. Random Forest remained the best-performing algorithm in the sensitivity analysis, with an AUC of 0.815 compared with 0.852 for the full model, corresponding to an absolute decrease of 0.037. Performance results for all six reduced models are reported in Supplementary Table S5.

**Table 2:** Testing-set performance of six machine-learning models for predicting DRG-related financial risk.

Model	Accuracy	F-beta score	AUC	Sensitivity	Specificity	AUPR
RF	0.768	0.772	0.852	0.786	0.751	0.837
LightGBM	0.761	0.764	0.841	0.776	0.748	0.816
KNN	0.757	0.741	0.818	0.698	0.815	0.819
NB	0.651	0.715	0.756	0.873	0.432	0.744
SVM	0.724	0.722	0.807	0.717	0.731	0.806
XGBoost	0.724	0.728	0.814	0.740	0.710	0.777

### 4.4 Feature importance analysis using SHAP

**Figure 5:**
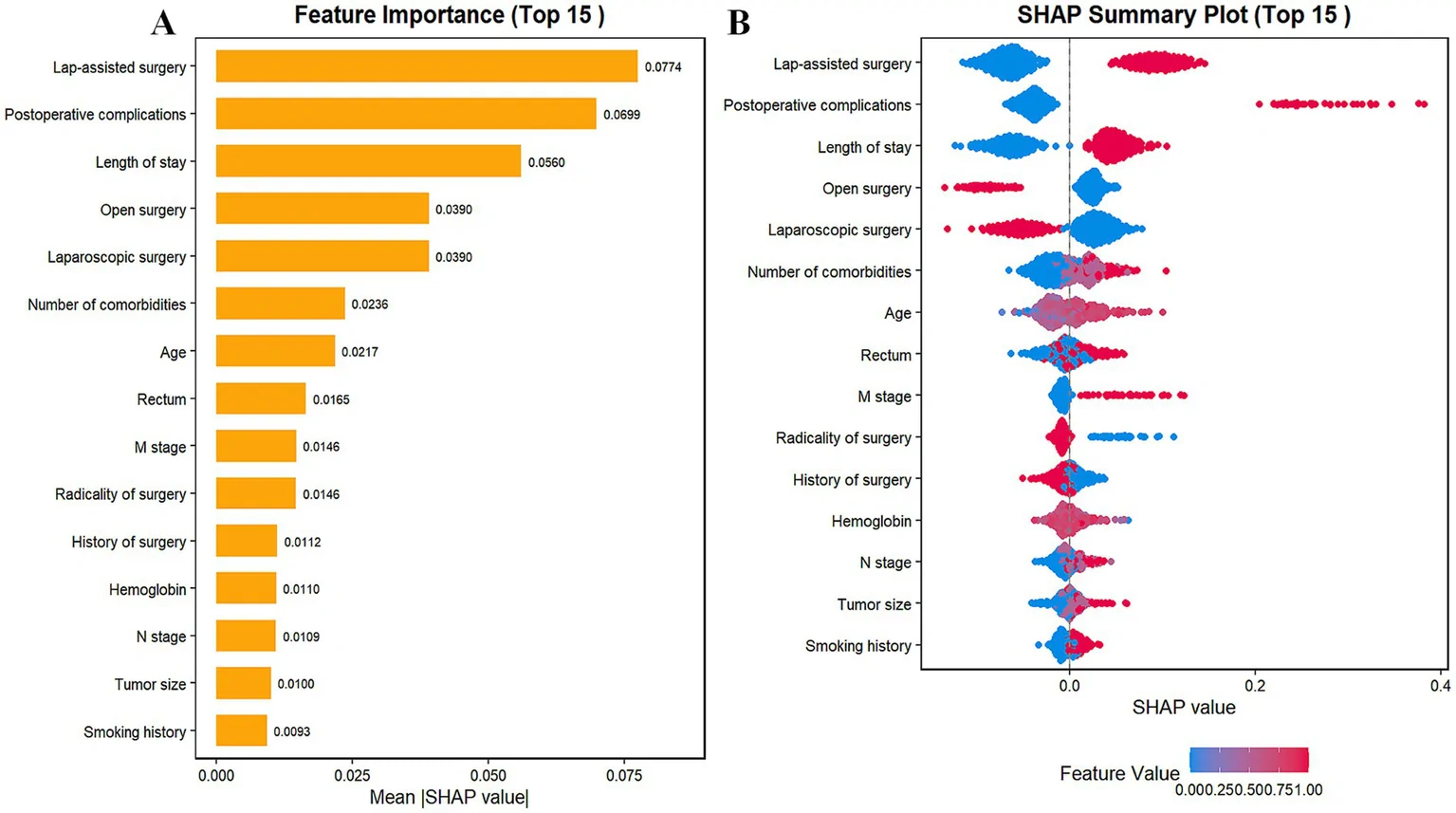
SHAP feature importance and summary analysis. **(A)** Mean absolute SHAP ranking of the top 15 features. **(B)** Beeswarm display of feature-value distribution and direction of effect on DRG-related financial risk.

The SHAP analysis was used to characterize the contribution of each feature in the optimal Random Forest model. Global importance in Figure 5A was ranked by mean absolute SHAP value. Surgical approach, particularly laparoscopy-assisted surgery, contributed most, followed by postoperative complications, length of stay, and comorbidity burden. These variables accounted for the main differences in predicted DRG-related financial risk. The prominence of surgical approach and complications indicates that both procedural factors and adverse perioperative events were central to the estimated financial deviation. The ranking was then used to guide directional and patient-level interpretation.

**Figure 6:**
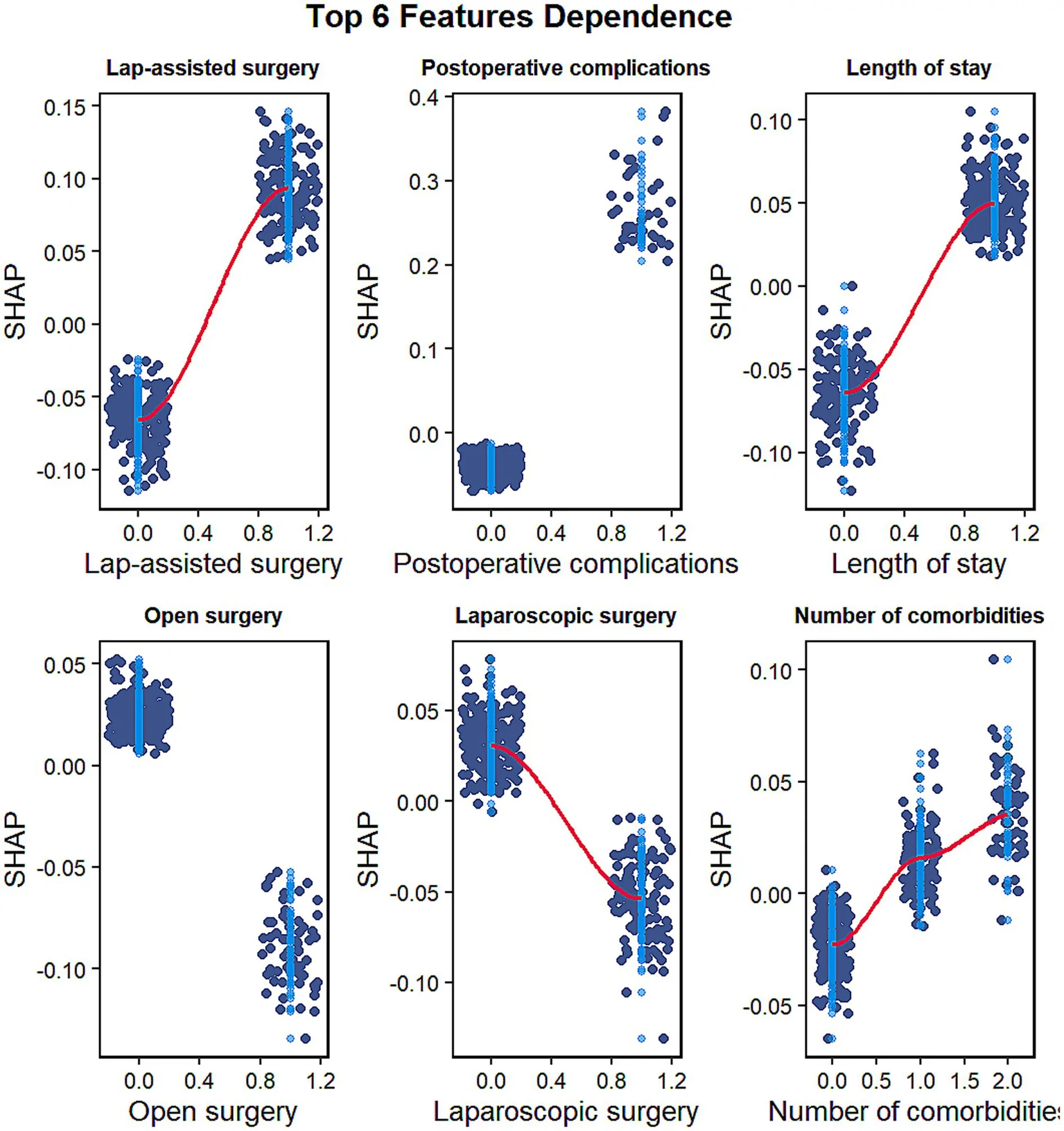
SHAP dependence plots for key continuous variables. Dependence plots showing non-linear associations and marginal effects for leading continuous predictors.

The beeswarm plot in Figure 5B further demonstrated the direction of these effects. Laparoscopy-assisted surgery and longer length of stay were located mainly on the positive SHAP axis, indicating increased estimated DRG-related financial risk. Postoperative complications showed the same positive direction. By contrast, open surgery and laparoscopic surgery were generally associated with negative SHAP values, indicating a lower probability of exceeding the DRG benchmark. SHAP dependence plots (Figure 6) were then used to examine non-linear effects and the ranges of continuous variables linked to changes in estimated DRG-related financial risk. Together, these analyses supplemented global importance with information on effect direction and functional form.

### 4.5 Patient-level explainability analysis

**Figure 7:**
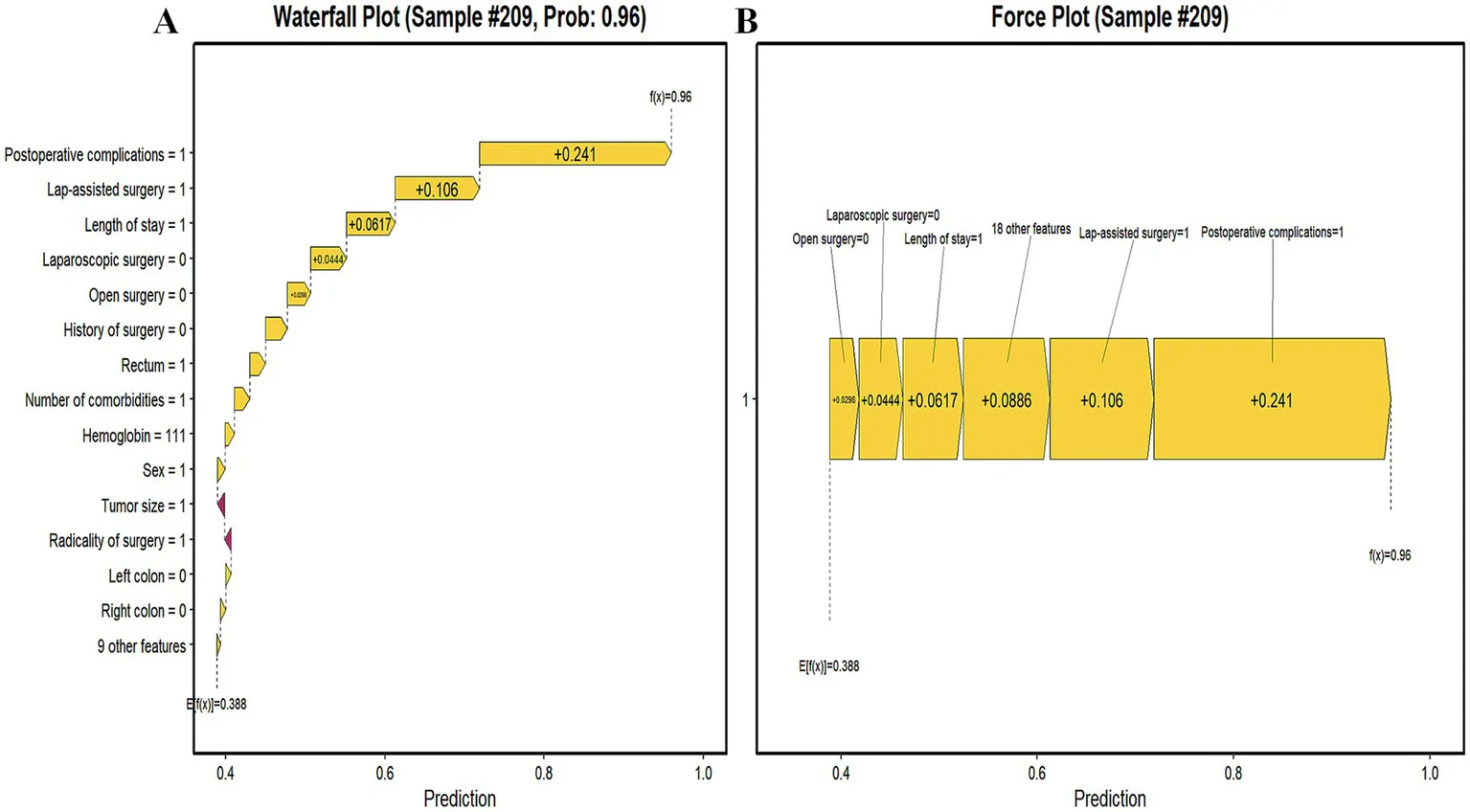
Patient-level SHAP analysis of DRG-related financial risk. **(A)** Waterfall plot of feature contributions to the prediction. **(B)** Force plot showing feature impacts on the individual prediction.

Patient-level SHAP visualizations were used to assess whether individual predictions could be explained in clinically meaningful terms. Figure 7 shows a representative high-risk patient (Sample #209). In the waterfall plot (Figure 7A), the prediction begins from the mean baseline risk (E[f(x)] ≈ 0.388). Postoperative complications, laparoscopy-assisted surgery, and prolonged length of stay all increased the estimate for this patient. Together, these contributions raised the predicted probability to 0.96 and placed the patient in the DRG-related financial-risk category. The visualization decomposed the difference between the cohort baseline and the individual estimate into specific feature contributions, allowing the high-risk result to be linked to perioperative events rather than presented as an unexplained model output.

### 4.6 Interactive online tool allows simulation of personalized interventions

**Figure 8:**
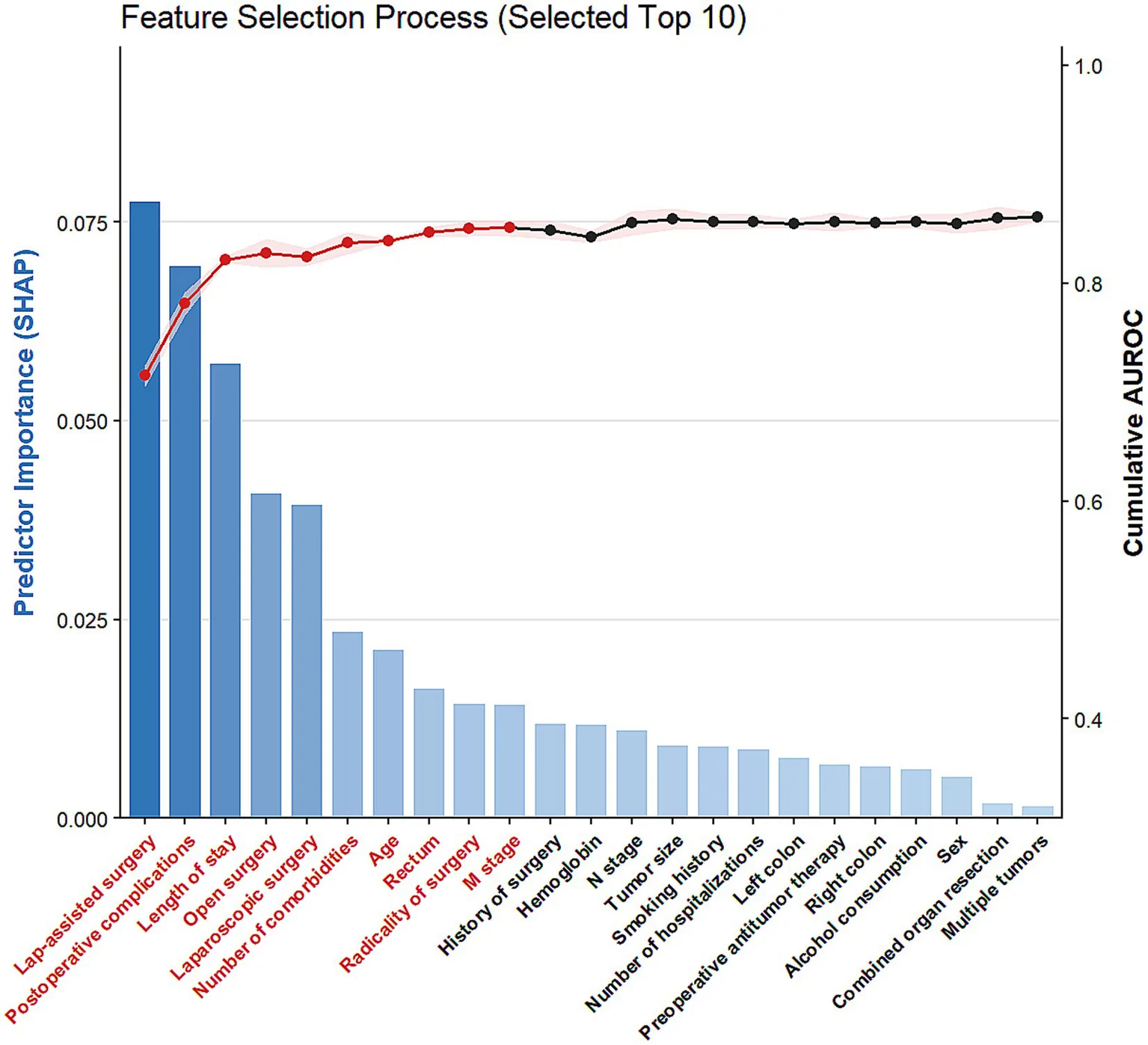
Sequential Forward Selection (SFS) and Model Parsimony. The curve tracks cumulative AUROC, identifying the top 10 features as the optimal subset where performance stabilizes.

**Figure 9:**
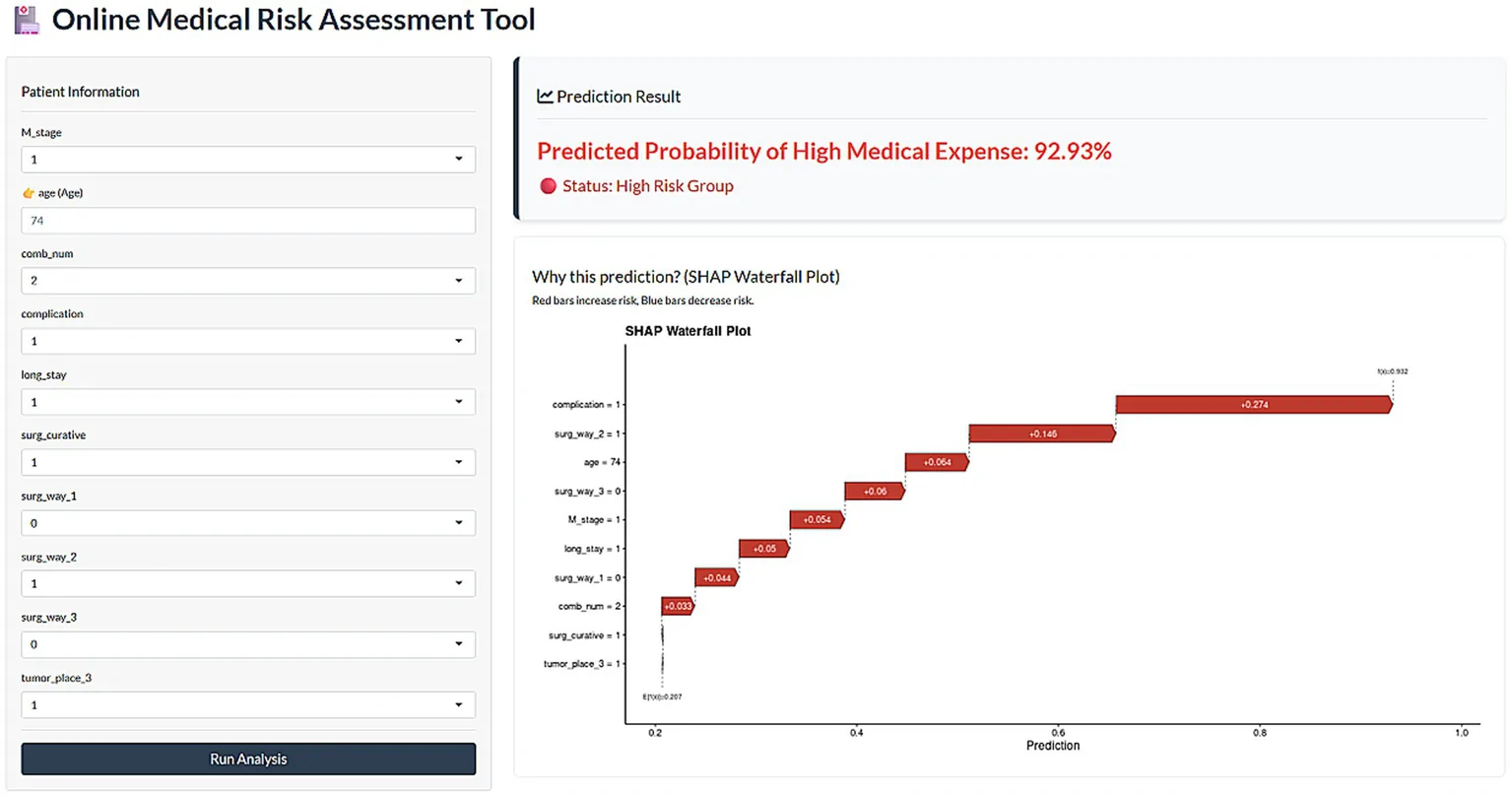
Interactive web-based risk calculator. Interface of the deployed tool for individualized risk assessment and perioperative scenario review (accessible at: https://clinical-ai-lab.shinyapps.io/Clinical-AI-Lab/).

For routine use, the full model was reduced to a smaller set of inputs. Sequential Forward Selection (SFS) showed a steep early rise in AUROC, followed by little additional improvement once the top 10 variables had been entered (Figure 8). Collecting more variables would therefore have added work for clinicians without a comparable gain in discrimination. We selected the Top 10 point and used it to construct the online calculator (Figure 9; available at https://clinical-ai-lab.shinyapps.io/Clinical-AI-Lab/). The interface reports an individual risk estimate and supports perioperative scenario review based on variables available across the care pathway. In this review, preoperative clinical factors, perioperative management targets, and downstream perioperative indicators such as length of stay and postoperative complications were interpreted according to their position in the clinical pathway. These simulations are not treatment recommendations. They are intended to make the relationship between a possible perioperative change and the model estimate easier to discuss.

**Figure 10:**
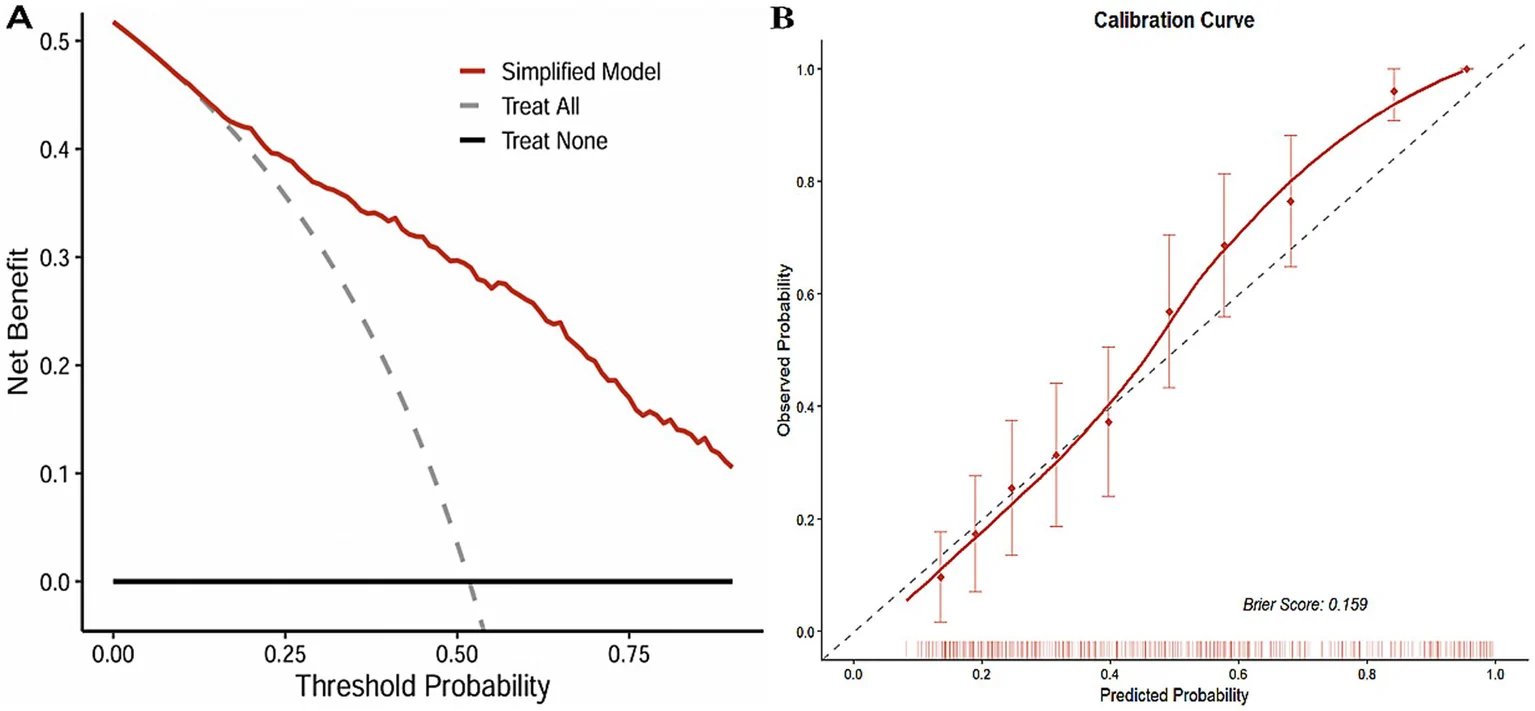
Clinical utility and calibration analysis. **(A)** Decision curve analysis (DCA): the red line indicates superior net benefit of the model-guided strategy over default alternatives. **(B)** Calibration curve: Plot showing high agreement between predicted and observed probabilities (Brier score = 0.159).

The calibration analysis and DCA addressed different questions about model use. In Figure 10B, predicted probabilities followed the observed event frequencies across the risk range. The Brier score was 0.159, which indicated relatively low overall prediction error and no obvious pattern of systematic overestimation or underestimation. DCA then considered whether using the estimate would improve decisions (Figure 10A). From threshold probabilities of 0.15–0.90, the model-guided strategy produced more net benefit than treating every patient or treating none. This result supports use of the model to identify patients for administrative review, including the “Special Case Negotiation” mechanism. This mechanism refers to case-by-case review and determination of reimbursement for necessary, complex care that falls outside conventional reimbursement policies. The model supplies evidence for that review; it does not determine the reimbursement decision or remove the need for expert assessment.

### 4.7 Prospective clinical feasibility evaluation

To examine the feasibility of the interactive web-based tool in a real-world perioperative workflow, we prospectively enrolled 12 inpatients at the Gastrointestinal Cancer Center of a tertiary hospital in Beijing. Routine preoperative risk assessments for DRG-related financial risk were conducted using the developed tool. Among the cohort, four patients were identified as having high DRG-related financial risk. The remaining eight patients were predicted to remain within the DRG benchmark, and postoperative cost records confirmed that their expenditures remained within the benchmark. Using the interactive tool, clinicians reviewed preoperative clinical factors, perioperative management targets, and downstream perioperative indicators, and observed the corresponding model-estimated change in DRG-related financial risk. The simulated changes in risk were reviewed by the clinical team when considering individualized perioperative management. Actual expenditure was verified from postoperative cost records. The following four clinical scenarios describe how the tool was used in patients initially identified as high risk. The corresponding SHAP visualizations are provided in the Supplementary Figures S5–S8. Records allowed changes to be compared with observed postoperative expenditure.

#### 4.7.1 Scenario 1: perioperative respiratory management

The first case involved a 70-year-old man with clinical stage cT3N1M0 rectal cancer, chronic bronchitis, emphysema, and a 30-year smoking history. Preoperative assessment estimated a 76.29% probability of DRG-related financial risk, mainly reflecting age, active smoking, and respiratory disease. Respiratory optimization was then considered using the calculator. The patient stopped smoking for 2 weeks and completed pulmonary exercises, including aerosol inhalation and sputum excretion. At reassessment, respiratory symptoms had improved and the estimated risk decreased to 21.94%. Surgery was completed without respiratory complications, and discharge occurred on postoperative day 6. Total expenditure was CNY 90,500, below the DRG benchmark. The paired estimates show how a preoperative intervention was reflected in the model before retrospective cost review.

#### 4.7.2 Scenario 2: strict glycemic control and surgical strategy

The second case involved a 60-year-old woman with cT4N1M0 sigmoid colon cancer and diabetes for 10 years. Her fasting blood glucose (FBG) was 10.2 mmol/L, raising concern for anastomotic leakage and prolonged hospitalization. The initial estimated probability of DRG-related financial risk was 74.62%. After endocrinology consultation, the antidiabetic regimen was adjusted and FBG decreased to 4.6 mmol/L. The repeated assessment estimated risk at 17.33%. Open surgery was selected, and the anastomosis was reinforced with barbed sutures. No major glycemic fluctuation or anastomotic leakage occurred during recovery. The patient was discharged 1 week after surgery, with total expenditure of CNY 92,300, within the DRG benchmark.

#### 4.7.3 Scenario 3: multidisciplinary management of cardiovascular comorbidities

The third case involved a 61-year-old obese woman with cT3N2M0 cancer of the hepatic flexure. She had a history of cardiac valve replacement and regular antiplatelet therapy, with a preoperative INR of 2.3. The combination of bleeding risk, thromboembolic risk, and perioperative medication adjustment led to an estimated 90.31% probability of DRG-related financial risk. After Multidisciplinary Team (MDT) review, weight management was initiated and antithrombotic treatment was changed to standardized bridging with low-molecular-weight heparin (LMWH). When INR decreased to 1.8, the estimate fell to 18.00%. Open surgery was performed with careful hemostasis, and antiplatelet treatment was restarted as planned. No bleeding or thrombotic event occurred. The patient was discharged on postoperative day 8, and total cost was CNY 98,000, within the DRG benchmark.

#### 4.7.4 Scenario 4: neoadjuvant therapy for advanced recurrence

The fourth case involved a 72-year-old man with recurrent sigmoid colon cancer at the previous anastomosis (cT4N3Mx), 2 years after his first operation. Although hypertension and diabetes were controlled, recurrent advanced disease and previous abdominal surgery increased operative complexity, including potential open conversion and postoperative bowel obstruction. The initial estimate of DRG-related financial risk was 63.57%. After neoadjuvant chemotherapy downstaged the tumor to ycT3N1M0, the estimate decreased to 43.82%. Open surgery was then performed, with placement of an anti-adhesion barrier to reduce postoperative complications. R0 resection was achieved, recovery was uncomplicated, and the patient was discharged on postoperative day 7. Total expenditure was CNY 94,500 and remained within the DRG benchmark. The two estimates reflected the change associated with tumor downstaging and the revised operative plan.

Across the four clinical scenarios, the tool was used to estimate how planned interventions might change financial outcomes and to support individualized management. By allowing selected scenario variables to be reviewed in real time, the interface made the cost implications of clinical decisions visible before the admission ended (Table 3). The estimates were used as decision support for each case, not as automatic instructions.

**Table 3:** Application of the interactive risk assessment tool in four initially high-risk clinical scenarios.

Patient	Primary risk features	Pre-intervention DRG-related financial risk	Post-intervention DRG-related financial risk	Implemented clinical interventions	Actual DRG benchmark status
Zhao	Advanced age, smoking, respiratory disease	76.29%	21.94%	Smoking cessation, pulmonary function exercises	Within benchmark
Li	Poorly controlled diabetes	74.62%	17.33%	Strict glycemic control, open surgery, meticulous anastomosis	Within benchmark
Chen	Obesity, history of cardiac valve replacement	90.31%	18.00%	MDT consultation, standardized bridging of anticoagulants/antiplatelets	Within benchmark
Sun	Advanced tumor stage, previous abdominal surgery	63.57%	43.82%	Neoadjuvant chemotherapy for tumor downstaging, intraoperative anti-adhesion barrier	Within benchmark

## 5 Discussion

The findings have practical relevance for DRG-based payment reform and value-based surgical oncology, particularly in settings where fixed reimbursement benchmarks need to be interpreted alongside case complexity. Standardized reimbursement promotes efficiency but may be insufficient for complex oncology care when legitimate resource use exceeds a fixed benchmark. Early identification of high-risk cases is therefore important for distinguishing expenditure related to clinical complexity from potentially avoidable resource use. This study developed and validated an interpretable machine learning framework for estimating DRG-related financial risk before final hospitalization cost was known. The framework was implemented as a point-of-care tool and prospectively examined in 12 patients during routine perioperative assessment. All enrolled patients underwent model-based risk assessment and postoperative expenditure verification; four initially high-risk cases further illustrated how patient-level explanations and repeated estimates were incorporated into clinical pathway review. By linking prediction, interpretability, deployment, and prospective workflow evaluation, the study adds an implementation-oriented component to CRC financial-risk research.

Random Forest provided the highest overall discrimination among the six algorithms (AUC = 0.852). For clinical and administrative use, the estimate also needed to be traceable to clinical features. SHAP explanations were therefore incorporated into the online calculator. The resulting output can support perioperative planning and provide structured information for administrative “Special Case Negotiation,” during which clinical and health insurance experts assess whether excess resource use reflects case complexity. The model does not classify expenditure as necessary or wasteful. It reports risk and contributing factors, leaving reimbursement decisions to expert review and the clinical record.

The modeling strategy was chosen to reflect the complexity of perioperative resource consumption. Linear regression, which has been used frequently in previous studies, assumes relatively separable associations between predictors and expenditure. In CRC surgery, patient demographics, tumor pathology, and treatment modalities may interact through non-linear and high-dimensional patterns. Random Forest was combined with Boruta feature selection and SMOTE class balancing to model these relationships. SHAP analysis was then used to make feature attribution explicit, addressing the limited transparency of ensemble models. This allowed the principal predictors to be assessed against established clinical pathophysiology rather than relying only on statistical discrimination.

Surgical approach was a principal determinant of predicted DRG-related financial risk (22). Laparoscopic surgery was identified as the second most important protective factor, consistent with evidence of reduced intraoperative bleeding, postoperative pain, time to intestinal recovery, and length of hospital stay (23, 24). Although minimally invasive surgery may require higher equipment expenditure, part of this cost may be offset by postoperative recovery benefits (25). Open surgery was also associated with lower predicted DRG-related financial risk in this cohort, possibly reflecting fewer advanced consumables, standardized perioperative routines, and lower direct procedural costs despite greater surgical trauma. These findings describe resource-use patterns in the study population and should not be interpreted as support for choosing an operative approach on financial grounds.

Laparoscopic-assisted surgery had the opposite association and represented the strongest risk factor for DRG-related financial risk. The approach often uses high-value consumables, including laparoscopic staplers, and may require an auxiliary open incision, thereby increasing instrument expenditure (26). It is also more commonly selected for technically difficult tumors or severe local adhesions, which can increase operative uncertainty and the likelihood of conversion to open surgery (27). In these cases, greater operative time and resource use may coincide with a recovery profile that does not fully match completely laparoscopic procedures. The association therefore likely reflects technique, consumable use, and case complexity rather than technique alone. Early financial-risk assessment may be particularly useful for laparoscopic-assisted cases.

Length of stay (LOS) was closely associated with predicted DRG-related financial risk, consistent with health economic evidence from other settings (28, 29). Additional hospital days require continued use of beds, staff time, medication, monitoring, and supportive services. In CRC surgery, prolonged admission may also indicate postoperative complications, infection, or delayed recovery (30). Comorbid disease can further increase resource use because several conditions may require consultation, investigation, and supportive treatment even without a single major complication (31). These results support complication prevention and appropriate implementation of Enhanced Recovery After Surgery (ERAS) protocols. They should not be interpreted as support for premature discharge, because LOS reduction is appropriate only when safe recovery is maintained.

Older age was associated with greater predicted DRG-related financial risk. Reduced physiological reserve may contribute to this association, as older patients can recover more slowly and may be more susceptible to postoperative morbidity (32). Hemoglobin (HGB) showed an inverse association. Higher HGB may reflect better nutritional and physiological reserve, whereas preoperative anemia in CRC has been associated with fatigue, delayed recovery, and transfusion requirements (33). The observed pattern is consistent with lower perioperative risk at higher HGB levels (34). Smoking remains clinically relevant because it is potentially modifiable. Through effects on tissue microcirculation, wound healing, and pulmonary complications, smoking may contribute to higher perioperative cost (35). These findings support preoperative assessment that separates fixed vulnerability from modifiable risk factors.

Advanced N and M stage increased predicted risk, consistent with the resource requirements of advanced disease. Such patients may require wider lymph-node dissection, combined organ resection, and neoadjuvant or adjuvant systemic treatment (36). Expenditure is therefore distributed across the treatment pathway rather than restricted to the index operation. The association also underscores the economic relevance of early detection. Earlier-stage diagnosis may permit less extensive intervention, improve prognosis, and reduce medical resource use (37). Tumor size showed a similar pattern, with larger tumors often requiring a wider operative field, greater technical effort, and more consumables.

The prospective cases illustrate the potential clinical use of the online tool. The web tool was used as a structured interface for reviewing how variables at different points in the perioperative pathway contributed to the estimated financial risk. Although DCA showed net benefit across a broad range of threshold probabilities compared with default strategies (38), implementation requires integration with clinical workflow. In the 12 prospective cases, clinicians interpreted the model estimate together with the patient’s condition and considered whether management of modifiable factors, such as comorbidity control or adherence to a clinical pathway, could alter risk. The risk estimate was then recalculated to support discussion of the revised plan. A lower simulated risk was not treated as evidence of guaranteed clinical success, and the calculator did not replace clinical judgment. Its role was to make potential resource consequences visible before retrospective financial review.

The model may also support administrative review for cases exceeding the standard reimbursement threshold. Narrative documentation is often used in such reviews but may not provide a standardized basis for distinguishing unavoidable cost from inefficient practice. By identifying clinical contributors to high expected cost, including advanced stage or severe comorbidity, the model provides structured evidence for supplemental reimbursement review. This evidence should be considered with the medical record and expert assessment. Used in this way, the tool may improve transparency and assist hospitals in managing complex cases without treating clinically necessary care as operational failure.

This study has limitations. First, the development cohort came from a single specialized cancer center, although it covered a decade of practice. Because reimbursement rules, prices, patient characteristics, and clinical pathways vary across institutions and regions, external validation is required before broader implementation. Because DRG payment rules evolved during the study period, the outcome reflects classification under a uniform current Beijing DRG 2.0 GB39 benchmark. Historical year-specific grouping rules and payment-policy changes were not reconstructed, and future validation in prospectively accrued patients managed under the current DRG framework is needed. Second, not all determinants of expenditure were captured. Surgeon experience, specific intraoperative events, and administrative processes may have contributed residual confounding. Third, the prospective evaluation was conducted in a single-center cohort of 12 patients and focused on workflow integration and patient-level application. Larger multicenter prospective studies should further evaluate model performance, clinical utility, and economic outcomes across institutions and reimbursement settings.

## 6 Conclusion

In conclusion, this study developed an interpretable machine learning approach for estimating DRG-related financial risk after colorectal cancer surgery. The model identified clinical contributors to risk and presented them through a point-of-care web tool. This may support earlier discussion of pathway adjustment and resource needs before expenditure is reviewed retrospectively. The same information may also inform exceptional reimbursement review when high cost reflects clinical complexity. The framework is designed to support clinical and administrative judgment rather than replace it. Further multicenter validation in routine workflows is needed before broader clinical or policy-level implementation.

## Data Availability

The datasets generated and analyzed during the current study are not publicly available due to the sensitive nature of patient clinical and financial information and the privacy regulations of the participating institution. De-identified datasets are available from the corresponding author (Zongjiu Zhang, zhangzongjiu@tsinghua.edu.cn) on reasonable request and with permission from the Medical Ethics Committee of Peking University Cancer Hospital & Institute. The interactive web-based tool is available at: https://clinical-ai-lab.shinyapps.io/Clinical-AI-Lab/.
