# Supplementary material for: Interpretable machine learning enables early identification of financial risk and resource optimization in colorectal cancer surgery
Source: Front Public Health. 2026 Aug 14;14:1918534. doi: 10.3389/fpubh.2026.1918534 (PMC13522193; doi:10.3389/fpubh.2026.1918534)
Supplement: Supplementary file 1 [file Supplementary_file_1.docx]

# Supplementary Materials

## S1 Table. Definitions and categories of variables presented in Table 1

| **Variable Group** | **Variable Name** | **Presentation in Table 1** | **Categories / Notes** |
| --- | --- | --- | --- |
| Patient Characteristics | Sex | Categorical | Male; Female |
|  | Age | Categorical | <55; 55-64; 65-74; ≥75 |
|  | Insurance Type | Categorical | Beijing urban; Beijing rural; Non-Beijing urban; Non-Beijing rural; Self-pay |
|  | Number of comorbidities | Categorical | 0; 1; ≥2. Comorbidities included hypertension, diabetes, heart disease, cerebrovascular disease, hepatitis, respiratory diseases, etc. |
|  | History of surgery | Categorical | Yes; No |
|  | Smoking history | Categorical | Yes; No |
|  | Alcohol consumption | Categorical | Yes; No |
|  | Drug allergy history | Categorical | Yes; No |
|  | Hemoglobin level | Categorical | <110 g/L; ≥110 g/L |
|  | Albumin level | Categorical | <35 g/L; 35-45 g/L; ≥45 g/L |
|  | Albumin/Globulin ratio (A/G) | Categorical | <1.0; 1.0-2.5; ≥2.5 |
| Disease Characteristics | Tumor location | Categorical | Right colon; Left colon; Rectum; Multiple sites |
|  | Pathological type | Categorical | Adenocarcinoma; Mucinous adenocarcinoma; Signet-ring cell carcinoma; Others |
|  | Differentiation | Categorical | Well; Moderate; Poor; Undifferentiated; Unknown |
|  | Tumor size (long diameter, cm) | Categorical | ≤3; 3-5; >5 |
|  | Multiple tumors | Categorical | Yes; No |
|  | T stage | Categorical | T0/Tis; T1; T2; T3; T4 |
|  | N stage | Categorical | N0; N1; N2; N3 |
|  | M stage | Categorical | M0; M1 |
| Treatment Characteristics | Preoperative antitumor therapy | Categorical | Yes; No |
|  | Number of hospitalizations | Categorical | 1; ≥2 |
|  | Length of hospital stay | Categorical | ≤10 days; >10 days |
|  | Postoperative complications | Categorical | Yes; No |
|  | Radicality of surgery | Categorical | Curative resection; Palliative resection |
|  | Surgical approach | Categorical | Open; Laparoscopy-assisted; Laparoscopic |
|  | Combined organ resection | Categorical | Yes; No |

Note: For model development, number of comorbidities, number of hospitalizations, and length of hospital stay were entered as categorical variables using the categories shown in this table.

## S2 Table. Definitions of Dependent Variables

| **Variable Name** | **Variable Type** | **Definition / Notes** |
| --- | --- | --- |
| Total cost | Continuous | Total in-hospital expenditure of the patient |
| Diagnosis cost | Continuous | Includes ultrasound, pathology, radiology, laboratory fees, etc. |
| Consumables cost | Continuous | Includes disposable medical materials for examination, treatment, surgery, and interventional procedures |
| Medication cost | Continuous | Includes Western medicine, antibiotics, albumin products, traditional Chinese medicine, etc. |
| Management cost | Continuous | Includes monitoring and auxiliary respiratory equipment, oxygen, bed fees, etc. |
| Medical service cost | Continuous | Includes consultation, surgery, anesthesia, general examination and treatment, interventional and rehabilitation treatment fees |
| Nursing cost | Continuous | Costs for nursing services provided to the patient |

## S3 Table. Annual CPI Ratios and Conversion of Historical Expenditure to 2024 Price Levels

| **Year** | **Annual CPI (previous year = 100)** | **Original Cost** | **Adjusted Cost Calculation** |
| --- | --- | --- | --- |
| 2024 | 99.80 | C1 | C1’ = C1 |
| 2023 | 100.20 | C2 | C2’ = C2 * 99.80% |
| 2022 | 100.70 | C3 | C3’ = C3 * 99.80% * 100.20% |
| 2021 | 99.80 | C4 | C4’ = C4 * 99.80% * 100.20% * 100.70% |
| 2020 | 104.90 | C5 | C5’ = C5 * 99.80% * 100.20% * 100.70% * 99.80% |
| 2019 | 108.40 | C6 | C6’ = C6 * 99.80% * 100.20% * 100.70% * 99.80% * 104.90% |
| 2018 | 103.00 | C7 | C7’ = C7 * 99.80% * 100.20% * 100.70% * 99.80% * 104.90% * 108.40% |
| 2017 | 107.40 | C8 | C8’ = C8 * 99.80% * 100.20% * 100.70% * 99.80% * 104.90% * 108.40% * 103.00% |
| 2016 | 102.60 | C9 | C9’ = C9 * 99.80% * 100.20% * 100.70% * 99.80% * 104.90% * 108.40% * 103.00% * 107.40% |
| 2015 | 100.20 | C10 | C10’ = C10 * 99.80% * 100.20% * 100.70% * 99.80% * 104.90% * 108.40% * 103.00% * 107.40% * 102.60% |
| 2014 | 99.90 | C11 | C11’ = C11 * 99.80% * 100.20% * 100.70% * 99.80% * 104.90% * 108.40% * 103.00% * 107.40% * 102.60% * 100.20% |

**Note: The annual CPI represents the price level in a given year relative to the preceding year, which is set to 100. A value of 100.20 therefore indicates a 0.20% increase from the preceding year. C1-C11 denote the original expenditures recorded from 2024 to 2014, respectively, and C1′-C11′ denote the corresponding expenditures expressed at 2024 price levels. For example, expenditure recorded in 2022 was converted as C3′ = C3 × (100.20/100) × (99.80/100), using the annual CPI ratios for 2023 and 2024.**

## S4 Table. Correlation Coefficients Between Variables and DRG-Related Financial Risk

| **Variable** | **Correlation Coefficient** | **Variable** | **Correlation Coefficient** |
| --- | --- | --- | --- |
| Sex | 0.077** | A/G ratio | -0.063** |
| Age | 0.115** | Tumor location | 0.126** |
| Insurance type | -0.026 | Pathological type | -0.024 |
| Hypertension | 0.123** | Differentiation | 0.015 |
| Diabetes | 0.069** | Tumor size (long diameter) | -0.003 |
| Heart disease | 0.068** | Multiple tumors | 0.048* |
| Cerebrovascular disease | 0.039 | T stage | 0.056* |
| Hepatitis | 0.030 | N stage | 0.080** |
| Respiratory disease | 0.049* | M stage | 0.142** |
| Number of comorbidities | 0.150** | Preoperative antitumor therapy | 0.091** |
| History of surgery | -0.004 | Number of hospitalizations | 0.110** |
| Smoking history | 0.090** | Length of hospital stay | 0.267** |
| Alcohol consumption | 0.033 | Postoperative complications | 0.328** |
| Drug allergy history | -0.009 | Radicality of surgery | 0.071** |
| Hemoglobin (HGB) | 0.008 | Surgical approach | -0.022 |
| Albumin (Alb) | -0.064** | Combined organ resection | 0.053* |

*P < 0.05, **P < 0.01

S5 Table. Sensitivity analysis of model performance after excluding postoperative complications and length of hospital stay

| **Model** | **Accuracy** | **F-beta score** | **AUC** | **Sensitivity** | **Specificity** | **AUPR** |
| --- | --- | --- | --- | --- | --- | --- |
| RF | 0.740 | 0.718 | 0.815 | 0.672 | 0.806 | 0.844 |
| LightGBM | 0.726 | 0.713 | 0.800 | 0.690 | 0.761 | 0.834 |
| KNN | 0.696 | 0.703 | 0.755 | 0.730 | 0.662 | 0.737 |
| NB | 0.632 | 0.550 | 0.702 | 0.491 | 0.772 | 0.661 |
| SVM | 0.698 | 0.692 | 0.780 | 0.691 | 0.704 | 0.784 |
| XGBoost | 0.708 | 0.694 | 0.781 | 0.672 | 0.744 | 0.819 |

This sensitivity analysis excluded postoperative complications and length of hospital stay from the candidate predictor set to evaluate model performance without these proximal resource-use indicators.

## ****S1 Fig. Feature preprocessing for machine learning analysis using SMOTE for synthetic oversampling.****

## S2 Fig. Precision-recall analysis of six machine learning models for predicting DRG-related financial risk. (A) Precision-recall curves. (B) Area under the precision-recall curves (AUPR).

## ****S3 Fig.**** Accuracy comparison of 6 machine learning models (A) and F-beta comparison of 6 machine learning models (B).

**（A）** **（B）**

## ****S4 Fig.**** Sensitivity comparison of 6 machine learning models (A) and Specificity comparison of 6 machine learning models (B).

**（A）** **（B）**

S5 Fig. SHAP visualization of Scenario 1 before and after simulated intervention.

S6 Fig. SHAP visualization of Scenario 2 before and after simulated intervention.

S7 Fig. SHAP visualization of Scenario 3 before and after simulated intervention.

S8 Fig. SHAP visualization of Scenario 4 before and after simulated intervention.
